# Validation of the Chinese version of the Yale-Brown Obsessive Compulsive Scale for Pathological Gambling for individuals with gambling disorder in Mainland China

**DOI:** 10.3389/fpsyt.2025.1535332

**Published:** 2025-05-29

**Authors:** Dongli Fan, Xiyuan Zhang, Siyao Shi, Jingyang Liu, Yicheng Wei, Ge Song, Yiyi Zhou, Yuanyuan Zhu, Jingyun Shi, Xingshi Cai, Gangliang Zhong, Jiang Du

**Affiliations:** ^1^ Shanghai Mental Health Center, Shanghai Jiao Tong University School of Medicine, Shanghai, China; ^2^ Department of Substance Dependence, Third People's Hospital of Fuyang, Fuyang, China; ^3^ Department of Psychiatry, The Affiliated Brain Hospital of Nanjing Medical University, Nanjing, Jiangsu, China

**Keywords:** Yale-Brown Obsessive Compulsive Scale, gambling disorder, validity, reliability, pathological gambling

## Abstract

**Introduction:**

The escalating severity of gambling issues in China highlights the need for culturally adapted assessment tools. The Yale-Brown Obsessive Compulsive Scale for Pathological Gambling (PG-YBOCS) is recognized for its ability to assess both the severity and compulsive features of gambling disorder.

**Methods:**

Given its emphasis on the compulsive features of gambling disorder, this study aimed to validate the Chinese version (PG-YBOCS-C) in clinical assessment. The 10-item PG-YBOCS-C was developed through translation and expert review. A total of 116 individuals with gambling disorder were recruited.

**Results:**

Reliability was assessed using Cronbach’s α coefficient (0.958) and test-retest reliability (0.722). The content validity index was 0.912, with item-level indices ranging from 0.778 to 1.000. Bartlett’s test of sphericity yielded X2 = 1123.86, P<0.001, and the KMO measure was 0.93. Exploratory factor analysis identified a single principal component accounting for 72.8% of the variance.

**Discussion:**

The reliability and validity of PG-YBOCS-C have been demonstrated, establishing it as a dependable tool for evaluating the severity of gambling symptoms in Chinese individuals.

## 1 Introduction

Gambling Disorder (GD), according to the DSM-5, is characterized by persistent and recurrent gambling behavior causing significant psychological distress or social impairment (1). The World Health Organization (WHO) reports that approximately 0.1% to 6% of adults worldwide suffer from this disorder (2). GD affects mental health, causing anxiety, depression, and suicidal ideation. It also leads to financial crises, social isolation, family breakdowns, and criminal behavior, increasing public health burdens and social instability (3).

According to a 2021 report by the United Nations Office on Drugs and Crime (UNODC), the illegal sports betting market sees annual wagers of up to $1.7 trillion (UN 4). This figure unequivocally highlights the significant risks and potential harms associated with gambling. Additionally, internet gambling has proliferated with the rapid development of the digital age due to its convenience, interactivity, and anonymity. It has become the fastest-growing mode of gambling, fundamentally changing how gamblers participate and making gambling behavior more difficult to regulate and control (5, 6).

In China, small-scale gambling is often seen as a form of entertainment deeply rooted in cultural traditions. In recent years, the prevalence of gambling has surged, surpassing levels seen in many other countries, and this situation has been further exacerbated by the rise of online gaming, which has become the dominant form of gambling in China (7). Despite laws prohibiting all forms of gambling, societal tolerance remains high, contributing to the popularity of gambling, particularly during major festivals (3, 8). However, social acceptance of gambling does not extend to an understanding of GD, which is often stigmatized and attributed to moral failings (9). This complex legal and cultural context leads to individuals with GD hiding their problems and being reluctant to seek help due to fear of legal consequences and stigma (10). Studies credited by Slutske (11) indicated that only 10% of individuals with GD seek treatment.

Assessing the severity of gambling is crucial for the treatment of GD, helping to determine individual risk levels and formulate targeted treatment plans. Currently, various scales for measuring GD have been developed internationally. Among these, self-report scales such as the Canadian Problem Gambling Index (CPGI) and the South Oaks Gambling Screen (SOGS) are widely used (12). However, these tools primarily rely on self-reports. In clinical studies, self-reported outcomes from individuals with GD may be biased due to their awareness of having received an intervention, particularly in terms of reported gambling behaviors and perceptions. Conversely, outcomes measured by an independent, blinded professional would have a lower risk of bias when assessing treatment effect (13). Additionally, individuals with GD may underestimate or deny the severity of their problems due to social pressure or distorted self-perception (14). Studies have shown that the self-reports of people with GD often indicate lower severity than those observed by third parties (10).

Observer rating scales, such as the Yale-Brown Obsessive Compulsive Scale for Pathological Gambling (PG-YBOCS), may provide a more objective method of assessment, combining observation and questioning, which may increase diagnostic reliability when examining treatment effects (15, 16). Moreover, GD shares significant neurocognitive characteristics with obsessive-compulsive disorder (OCD), including ritualized behaviors, intrusive thoughts, and diminished impulse control (16). The PG-YBOCS developed by Pallanti et al. (16) specifically targets gambling-related thoughts and behaviors, measuring the severity of obsessive-compulsive disorder, especially for obsessions and compulsions. Multiple studies confirmed that PG-YBOCS can effectively distinguish different severity levels of GDs and has been widely used as a primary measure of treatment efficacy (17–20). PG-YBOCS has been translated into several languages, such as Japanese (21), demonstrating its cross-cultural applicability and importance in global addiction research.

In China, several self-report tools for GD exist, including the Chinese Version of the Canadian Problem Gambling Index, the South Oaks Gambling Screen and the Chinese Gambling Urge Scale (22–24). An observer rating scale administered by a professional, such as a psychologist or a clinical tester in clinical assessment has not been developed. Given the gap in GD assessment, the objective and standardized PG-YBOCS can effectively measure the severity of GDs. Localizing and validating the Chinese Version of PG-YBOCS (PG-YBOCS-C) is crucial for assessing and treating GDs in China, promoting clinical research in this field.

## 2 Methods

### 2.1 Participants

From September 2023 to June 2024, 116 male individuals with GD in the Department of Addiction Treatment at Shanghai Mental Health Center in Mainland China were recruited. The average age was 30.63 years (SD = 6.33, range: 22-55), the average education duration was 14.69 years (SD = 6.88, range: 6-19), and the average duration of gambling was 7.72 years(SD = 4.97, range: 1-30).

Inclusion criteria: Individuals who met the diagnostic criteria for GD in DSM-5 (≥4); had sufficient cognitive ability to complete all assessments and related clinical examinations required for this study; and exhibited normal hearing and vision.

Exclusion criteria: IQ < 70; had taken medications that promoted cognitive functions in recent months; had other physical diseases or cognitive impairments besides GD, such as schizophrenia; other situations deemed unsuitable for participation in the study by the researchers.

### 2.2 Procedure

The adaptation of PG-YBOCS for the Chinese context involved a rigorous and meticulous process to ensure linguistic accuracy and cultural relevance. The PG-YBOCS-C was translated from the original scale into Chinese by two experts in GD, and the initial Chinese version was formed after review. Subsequently, a bilingual expert who had not previously been exposed to the original scale translated the initial Chinese version into English to ensure translation accuracy. An expert committee then reviewed the translation to ensure content and conceptual equivalence between the original and the Chinese version. To validate and refine the scale, the initial Chinese version was first pretested on 30 individuals with GD, and revisions and cross-cultural wording adjustments were made based on feedback. Ultimately, after multiple rounds of correction and optimization, the PG-YBOCS-C used in this study was finalized.

Individuals with GD who met the experimental criteria were required to complete the assessment. Before the formal assessment, they read and signed the informed consent form and completed the PG-YBOCS-C rating by others as well as other related questionnaires under the guidance of trained professional assessors. The PG-YBOCS-C assessment took approximately 10 minutes, conducted through interviews with each individual by professional assessors, without direct contact with the test items by the individuals. Other questionnaires were completed by themselves. The PG-YBOCS-C retest rating by others was completed two weeks later through telephone interviews conducted by professional assessors. All assessors received standardized training in PG-YBOCS-C interview techniques by experienced clinicians, using a scoring manual to ensure consistency in administration and rating. In total, 55 individuals with GD completed the retest.

### 2.3 Measures

The evaluation scores of the Gambling Symptom Assessment Scale (G-SAS) for GD reflect the specific severity of GD, which is consistent with the measurement direction of the rating tool aimed to be revised in this study. The score of the Gambling Eagerness Scale (GES) indicates the level of immediate gambling eagerness. Therefore, the G-SAS and the GES for GD are used as the primary criterion for measurement in this study. Additionally, GD has significant associations with traits such as motor impulsivity, decision-making impulsivity, and individual self-control ability (25). Therefore, this study also measured the Barratt Impulsiveness Scale (BIS-11) and the Chinese Version of the Self Control Scale (SCS) as criteria (26).

#### 2.3.1 DSM-5 criteria for gambling disorder

The DSM-5 provides a comprehensive diagnostic framework for identifying GD. According to the assessment standards of DSM-5 (1), a score over 4 was considered indicative of GD. The total score ranged from 4 to 9, with 1 point assigned for meeting each diagnostic criterion. A higher score indicated more pronounced manifestations and severe symptoms of GD. Individuals who meet the diagnostic criteria for GD will be included in this study. DSM-5 is regarded as the gold standard for diagnosing GD due to its high reliability and validity (27).

#### 2.3.2 Pathological Gambling Adaptation of the Yale-Brown Obsessive Compulsive Scale

The PG-YBOCS consists of 10 items of clinician-administered questions, primarily measuring gambling thoughts and impulses as well as gambling behaviors. The scale is divided into two sections: the first five items target gambling urges and thoughts while the remaining five focus on the behavioral component of the disorder. Both components assess key domains including time spent on gambling activities, functional impairment, emotional distress, resistance attempts, and perceived control over gambling behavior. It employs a Likert 5-point scale (0-4), assessing the severity of GD during the most recent time interval (usually within the past week). Each question is scored separately, with a total score obtained by summing both. A higher total score indicates a more severe level of GD. The severity is classified as subclinical, mild, moderate, severe, and extreme levels of GD according to the score. Specific scale items are presented in **Table 1**. Previous research has shown that the Japanese version of PG-YBOCS has good reliability and validity in clinical samples, with a Cronbach’s α of 0.85 (Yokomitsu et al., 2020).

**Table 1:** The specific items of PG-YBOCS-C.

The following questions are about gambling thoughts and urges.
1. Time occupied by gambling thoughts/urges every day.
0 = None at all. 1 = Mild (less than 1 hour), or occasional (no more than 8 times a day). 2 = Moderate (1–3 hours), or frequent (more than 8 times a day, but not for most of the day). 3 = Severe (3–8 hours), or with a very high frequency (more than 8 times a day, and for most of the day). 4 = Extremely severe (more than 8 hours), or almost all the time.
2. Interference due to gambling thoughts/urges.
0 = No interference. 1 = Mild – Slightly interferes with social or work activities, but overall performance is not significantly affected. 2 = Moderate – Significantly interferes with social or work activities, but still manageable. 3 = Severe – Causes difficulties in social or work performance. 4 = Extreme – Unable to manage social or work activities.
3. Distress associated with gambling thoughts/urges.
0 = None. 1 = Mild – Slight distress. 2 = Moderate – Distressed but still manageable. 3 = Severe – Very distressed. 4 = Extreme – Distress is nearly constant and interferes with functioning.
4. Resistance against gambling thoughts/urges.
0 = Constantly resisting, or symptoms are so mild that no resistance is needed. 1 = Actively resisting most of the time. 2 = Makes some effort to resist. 3 = Gives in all gambling thoughts and urges without trying to resist, but still feels some reluctance. 4 = Completely gives in to gambling thoughts and urges.
5. Degree of control over gambling thoughts/urges.
0 = Complete control. 1 = Mostly able to control with some effort and attention; can stop or shift focus. 2 = Moderate control; “sometimes” able to stop or shift obsessive thoughts. 3 = Poor control; rarely able to successfully stop or disregard gambling thoughts or urges, finding it difficult to shift focus. 4 = No control; completely unable to stop or even shift attention away from gambling thoughts or urges.

The following questions are about gambling-related behaviors.
6. Time spent performing gambling-related behaviors every day.
0 = None at all. 1 = Mild (less than 1 hour a day), or occasional (infrequent occurrences). 2 = Moderate (1–3 hours a day), or frequent (occurs often but not for most of the day). 3 = Severe (3–8 hours a day), or very frequent (occurs for most of the day). 4 = Extremely severe (more than 8 hours a day), or almost constantly.
7. Interference due to gambling-related behaviors.
0 = No interference. 1 = Mild – Slightly interferes with social or work activities, but overall functioning remains intact. 2 = Moderate – Significantly interferes with social or work activities, but still manageable. 3 = Severe – Causes significant difficulties in social or work performance. 4 = Extremely severe – Unable to cope with social or work activities.
8. Distress associated with gambling-related behaviors.
0 = None. 1 = Mild – Feels only slight anxiety if gambling-related behaviors are prevented. 2 = Moderate – Feels moderate but manageable anxiety when gambling-related behaviour is prevented. 3 = Severe – Feels significant distress and anxiety if gambling-related behaviors are prevented. 4 = Extremely severe – Experiences extreme anxiety if gambling-related behaviors are prevented.
9 .Resistance against gambling-related behaviors.
0 = Constantly resisting, or symptoms are so mild that no resistance is necessary. 1 = Actively resisting most of the time. 2 = Makes some effort to resist. 3 = Gives in to all gambling-related behaviors without attempting to resist, but still feels some reluctance. 4 = Completely gives in to gambling-related behaviors.
10. Degree of control over gambling-related behaviors.
0 = Complete control. 1 = Mostly able to control; with some effort and attention, can stop gambling-related behaviors. 2 = Moderate control; “sometimes” able to control gambling-related behaviors, but with some difficulty. 3 = Poor control; able to delay the behavior briefly, but eventually must carry it out. 4 = No control; unable even to delay gambling-related behaviors.

#### 2.3.3 Gambling Symptom Assessment Scale

As a self-report scale, G-SAS is designed to assess the severity of symptoms among GD (28). The scale consists of 12 items that measure gambling symptoms over one week, including gambling urges, gambling thoughts, and actual gambling behaviors. The total score ranges from 0 to 48, with higher scores indicating more severe symptoms. The score is defined as follows: a score below 8 indicates no gambling symptoms; a score of 8–20 indicates mild symptoms; a score of 21–30 indicates moderate symptoms; a score of 31–40 indicates severe symptoms; and a score above 40 indicates extreme gambling symptoms. The Cronbach’s α of G-SAS in this study was 0.926 (29).

#### 2.3.4 Gambling Eagerness Scale

The Gambling eagerness Scale is designed to measure the current level of gambling urges among individuals, requiring individuals with GD to rate their gambling urges based on their immediate feelings (30). This scale is a single-item rating scale ranging from 0 to 10, with 0 representing ‘no eagerness for gambling’ and 10 representing ‘extreme high eagerness for gambling’.

#### 2.3.5 Barratt Impulsiveness Scale Version 11

BIS-11, including 30 items, reflects individuals’ impulsivity levels and is used to assess the likelihood of impulse control disorders. A higher score indicates a greater likelihood of impulse control disorders and more pronounced impulsive characteristics. The Cronbach’s α of BIS-11 in this study was 0.761 (31).

#### 2.3.6 Self-control Scale

The SCS primarily assesses the ability of individuals with GD to exert self-control. The scale consists of 19 items and employs a Likert 5-point scoring system. A higher total score indicates a greater degree of self-control. Analysis in this study revealed that the Cronbach’s α of the SCS in this study was 0.671 (32).

### 2.4 Statistical analysis

IBM SPSS 23.0 was used for analysis. For item analysis, individuals were ranked in descending order with the top 27% and bottom 27% classified into high and low score groups, respectively. An independent samples t-test was employed to determine the cut-off value. The internal consistency reliability of the scale was assessed by Cronbach’s α, while the test-retest reliability over two weeks was determined through Pearson’s correlation coefficient. Additionally, the content validity of the scale was evaluated by expert consultation at both the item and scale levels. Exploratory factor analysis was conducted to identify the factor structure of the PG-YBOCS-C, with the Kaiser Meyer Olkin (KMO) measure and Bartlett’s test of sphericity ensuring the suitability of factor analysis. The principal component analysis method and varimax rotation were used to explore the factor structure of the scale.

## 3 Results

### 3.1 Item analysis

The item analysis revealed that the critical values (CR) of each item ranged from 13.16 to 25.36, with significant differences in scores between the high score and low score groups across all items (*P* < 0.001). In addition, the Pearson correlation analysis was used to analyze the correlation between individual item scores on the PG-YBOCS-C and the total score. The results showed that each item score was positively correlated with the total scale score at a significant level (**Table 2**).

**Table 2:** Item analyses and correlations of the PG-YBOCS-C.

Items		Critical Value (CR)	*r*
1	Time occupied by gambling thoughts/urges	22.10**	.90**
2	Interference due to gambling thoughts/urges	20.36**	.86**
3	Distress associated with gambling thoughts/urges	16.90**	.84**
4	Resistance against gambling thoughts/urges	13.16**	.80**
5	Degree of control over gambling thoughts/urges	19.13**	.85**
6	Time spent performing gambling-related behaviors	25.36**	.86**
7	Interference due to gambling-related behaviors	15.83**	.86**
8	Distress associated with gambling-related behaviors	14.94**	.80**
9	Resistance against gambling-related behaviors	16.67**	.86**
10	Degree of control over gambling-related behaviors	24.34**	.90**

**P<.001.

PG-YBOCS-C, the Chinese Version of the Yale-Brown Obsessive Compulsive Scale for Pathological Gambling.

### 3.2 Reliability analysis

For reliability analysis, the PG-YBOCS-C exhibited strong internal consistency overall with a Cronbach’s α of 0.958. The Cronbach’s α values for each item are detailed in **Table 3**. Additionally, test-retest reliability was assessed over a two-week interval with a subset of 55 participants. The test-retest reliability of the PG-YBOCS-C was 0.722, with *P*<0.01, which is statistically significant. The result indicates that the PG-YBOCS-C scale exhibits good stability across time, demonstrating high consistency and reliability.

**Table 3:** Result of internal consistency and exploratory factor analysis.

Item		Cronbach’s α if item deleted	Factor loading
1	Time occupied by gambling thoughts/urges	.951	.905
2	Interference due to gambling thoughts/urges	.953	.864
3	Distress associated with gambling thoughts/urges	.955	.798
4	Resistance against gambling thoughts/urges	.956	.834
5	Degree of control over gambling thoughts/urges	.954	.860
6	Time spent performing gambling-related behaviors	.953	.859
7	Interference due to gambling-related behaviors	.953	.794
8	Distress associated with gambling-related behaviors	.956	.850
9	Resistance against gambling-related behaviors	.953	.862
10	Degree of control over gambling-related behaviors	.951	.901

### 3.3 Validity analysis

For content validity, the item content validity index (ICVI) and the scale content validity index (SCVI) were evaluated. Nine experts in psychology and psychiatry were invited to rate the appropriateness, comprehensibility, and representativeness of each item on the scale using 5-point scale (1 = very poor, 5 = very good). The ICVI was calculated as the proportion of experts who rated an item as 5, divided by the total number of experts, while the SCVI was derived as the mean of the experts’ ratings. The results showed that ICVI ranged from 0.778 to 1. The SCVI of the PG-YBOCS-C was 0.912, indicating a good content validity of PG-YBOCS-C.

Regarding structural validity, the result indicated that the scale was suitable for subsequent factor analysis (KMO=0.95, Bartlett’s Test of Sphericity X^2^ = 1123.86, *P*=0.000<0.01). Based on this, exploratory factor analysis was performed on 116 valid datasets using principal component analysis and varimax rotation, with factors extracted based on eigenvalues greater than 1. This analysis revealed that the PG-YBOCS-C has a unidimensional structure, explaining 72.811% of the total variance. All item factor loadings exceeded 0.4, suggesting strong factor loadings, with the factor loadings detailed in **Table 3**. These results demonstrate that the PG-YBOCS-C exhibits good structural fit and high structural validity.

Regarding criterion validity, correlation analysis between the PG-YBOCS-C and other variables(G-SAS, GES, SCS, BIS-11) was conducted. Results about indicators of GD severity showed that the PG-YBOCS-C was significantly correlated with the GES (*r*=.38, *p*<.01) and the G-SAS (*r*=.53, *p*<.01). Other indicators demonstrated a negative correlation between the PG-YBOCS-C and the SCS (*r*=-.32, *p*<.01), while a positive correlation was found with the BIS-11 (*r*=.31, *p*<.01) (**Table 4**).

**Table 4:** Means and standard deviations and correlation coefficients among them.

Scales	*M ± SD*	PG-YBOCS-C	GES	SCS	BIS-11	G-SAS
PG-YBOCS-C	14.91 ± 11.77	1				
GES	3.11 ± 3.04	.38^**^	1			
SCS	51.10 ± 12.39	-.32^*^	-.39^**^	1		
BIS-11	50.75 ± 12.45	.31^**^	.29^**^	-.50^**^	1	
G-SAS	12.59 ± 10.80	.53^**^	.38^**^	-.37^**^	.34^**^	1

^*^P<.05, ^**^P<.01.

PG-YBOCS-C, the Chinese Version of the Yale-Brown Obsessive Compulsive Scale for Pathological Gambling.

GES, the score of the Gambling Eagerness Scale.

SCS, the Chinese Version of the Self Control Scale.

BIS-11, Barratt Impulsiveness Scale Version 11.

G-SAS, Gambling Symptom Assessment Scale.

## 4 Discussion

To our knowledge, this is the first study to focus on the reliability and validity of an observer rating scale for GD in Chinese mainland. The results confirm that PG-YBOCS-C is a reliable tool for assessing symptom severity in Chinese individuals with GD. This tool effectively fills the gap in the current research on GDs, providing the instrument for clinical assessment, and will help clinical staff to evaluate and develop treatment plans.

In this study, item analysis revealed that PG-YBOCS-C have good discrimination, indicating significant power in differentiating individuals with varying symptom severity. The scale showed high internal consistency with a Cronbach’s α of 0.965. Each of the 10 items demonstrated good discriminatory ability, and all item scores correlated significantly with the total score, further validating the scale’s internal consistency. Test-retest reliability over two weeks showed a coefficient of 0.722, emphasizing the scale’s stability and repeatability. The content validity index (SCVI) was 0.912, confirming expert agreement on the relevance of these items to gambling symptoms. This highlights the scale’s applicability and professionalism in the field of gambling research, supported by its robust content validity. The findings are consistent with the findings of Yokomitsu et al. (2020) in Japan.

Many researchers have proposed that impulse control disorder is a core symptom of addictive behavior, including GD. Addictive behavior is often described as compulsive (33). Given the similarities between OCD and GD (16), particularly in terms of ritual behavior and the compelling urge to gamble, as well as the correlations between OCD-like symptoms and gambling severity (34), the Yale-Brown Obsessive-Compulsive Scale (Y-BOCS) (35) has been adapted for pathological gambling (PG-YBOCS) (16). Contrary to the two-dimensional structure of Y-BOCS (obsessive thoughts and compulsions) (36), Pallanti et al. (16) identified a unidimensional structure for the PG-YBOCS. This also proves that compulsive behaviors in OCD tend to be more complex and specific, often disconnected from direct impulses (37). Our findings align with this unidimensional model, indicating that addictive behavior is intrinsically linked to the urge itself, making the two inseparable (e.g. gambling behavior may intensify corresponding urges, creating a vicious cycle) (38).

To examine the effectiveness of PG-YBOCS-C in assessing the severity of GD, the G-SAS was employed, a tool that has been proven reliable and effective in measuring symptom severity and monitoring treatment progress (28). The significant positive correlations between PG-YBOCS-C and G-SAS indicated that PG-YBOCS-C is an effective measure of GD severity, as further evidenced by its significant positive correlation with a single-item GES scale measuring gambling eagerness. Furthermore, consistent with previous findings, the significant positive correlations between PG-YBOCS-C and BIS-11 suggested that higher impulsivity levels are associated with more severe GDs (25). The significant negative correlation between PG-YBOCS-C and SCS (39) suggested that individuals with low self-control are less able to inhibit impulses for immediate gratification, making them more susceptible to engaging in risky behaviors, including GDs.

This study highlighted the practicality and effectiveness of the PG-YBOCS-C in the Chinese cultural context, providing a reliable observer rating tool for assessing GD symptoms in Chinese clinical practice. It addressed the need for gambling assessment tools to be adapted to the unique social and cultural landscape (7). As a clinician-administered tool, it reduces the tendency to underreport gambling behaviors due to shame or bias, thereby enhancing the accuracy and reliability of assessments. Owing to its concise and structured format, the PG-YBOCS-C is well-suited for application in clinical triage, risk screening, and symptom monitoring. It can also be combined with other tools to provide a more comprehensive assessment (such as self-report scales).

Although this study validated PG-YBOCS-C as an effective clinical tool, there were some limitations of the study. First, despite efforts to recruit a broad participant base, the sample size remained relatively small, potentially limiting the generalizability of the results. Given the limited sample size, this study only conducted EFA to examine the structural validity of the scale. While the EFA results suggested a strong unidimensional structure explaining 72.8% of the total variance, the use of EFA alone may carry certain limitations, such as potential overfitting and reduced generalizability of the factor structure. Additionally, the sample consisted exclusively of men, which may lead to gender biases. Furthermore, as the sample was drawn solely from mainland China, the applicability of the findings to other regions, such as Hong Kong and Macau, may be constrained due to differing sociopolitical contexts. Even so, the results of this study still provided a favorable tool for clinical research in the area of GD in the Chinese Mainland. To enhance the diversity and representativeness of the findings, future studies should include more diverse samples from various regions.

## 5 Conclusion

In conclusion, the results of the present study indicate that the PG-YBOCS-C is a reliable and valid measure for assessing the severity of GD in the Chinese mainland. Our findings validate that the scale is a reliable observer rating tool for assessing the severity of gambling symptoms in these individuals.

## Data Availability

The raw data supporting the conclusions of this article will be made available by the authors, without undue reservation.
